# Supplementary figures and images for: Uricase-deficient rat is generated with CRISPR/Cas9 technique
Source: PeerJ. 2020 Apr 27;8:e8971. doi: 10.7717/peerj.8971 (PMC7192158; doi:10.7717/peerj.8971)

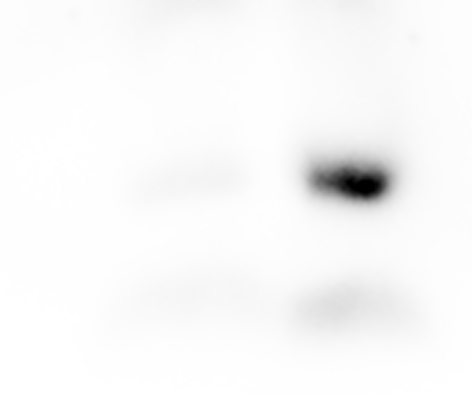

Supplement: Supplemental Information 2 [file peerj-08-8971-s002.png]

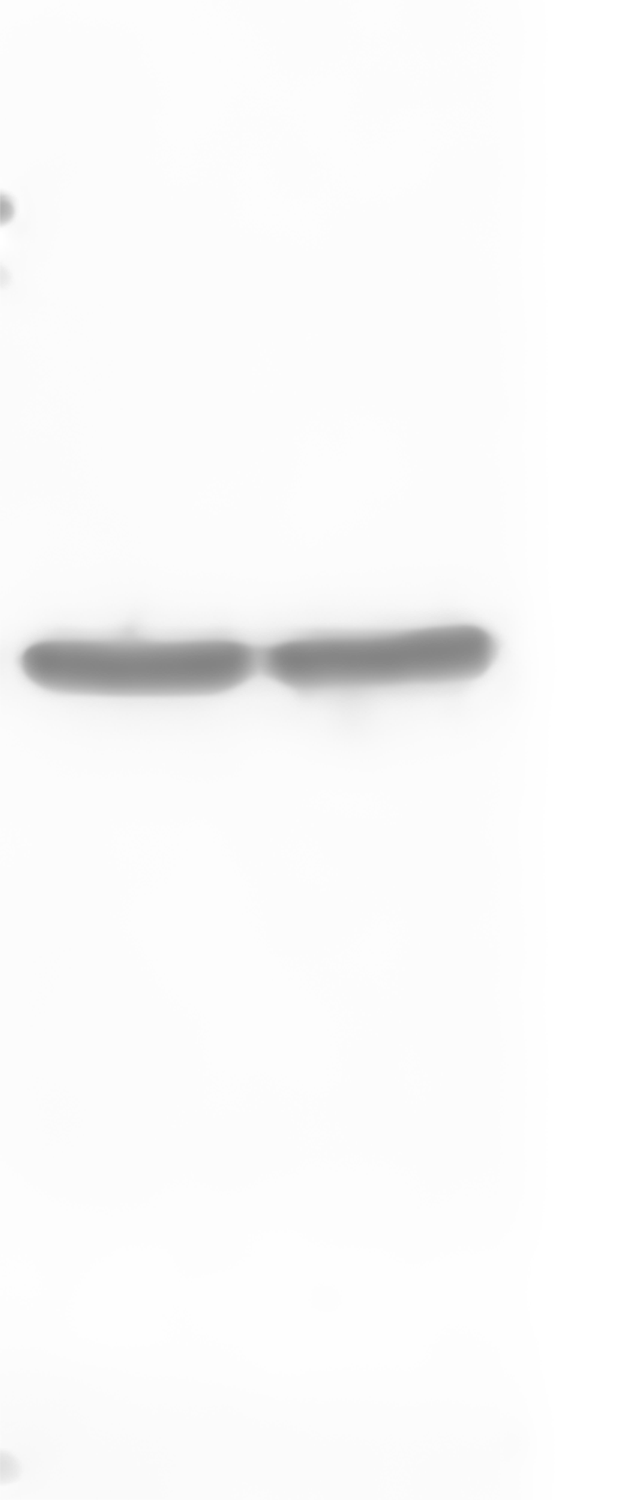

Supplement: Supplemental Information 3 [file peerj-08-8971-s003.png]

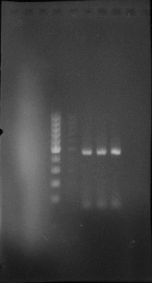

Supplement: Supplemental Information 4 [file peerj-08-8971-s004.png]

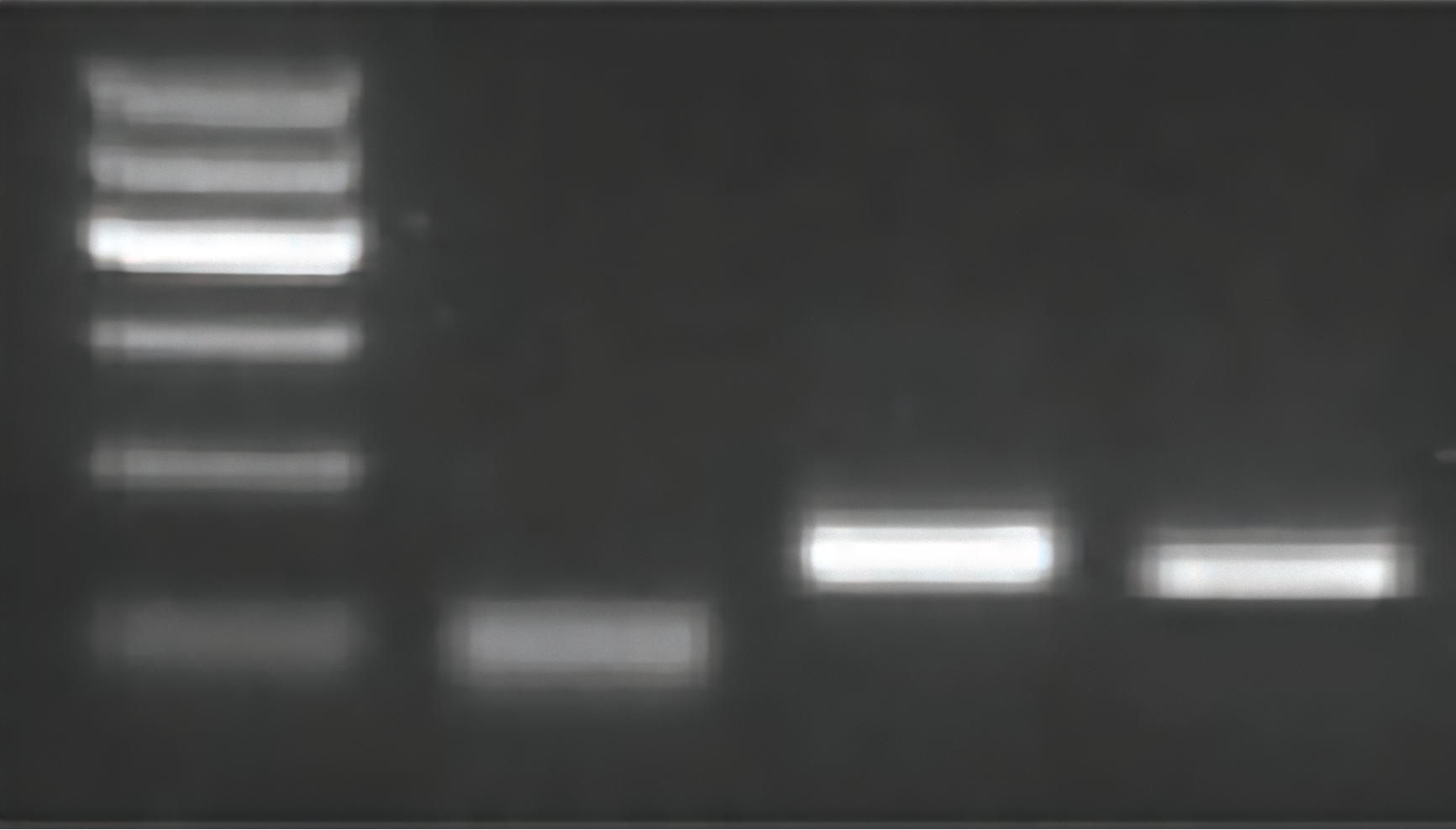

Supplement: Supplemental Information 5 — None [file peerj-08-8971-s005.png]

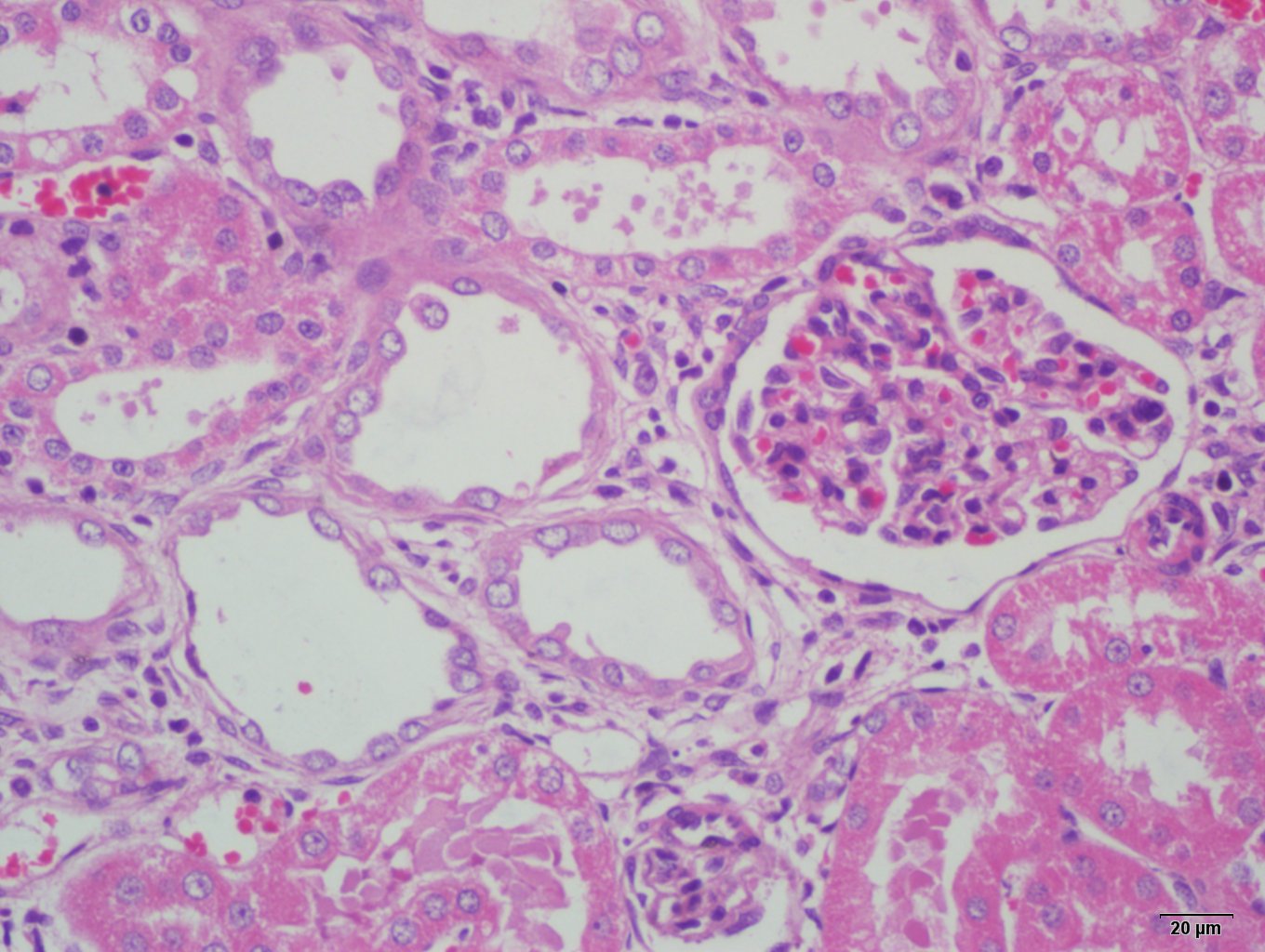

Supplement: Supplemental Information 19 [file peerj-08-8971-s019.jpg]

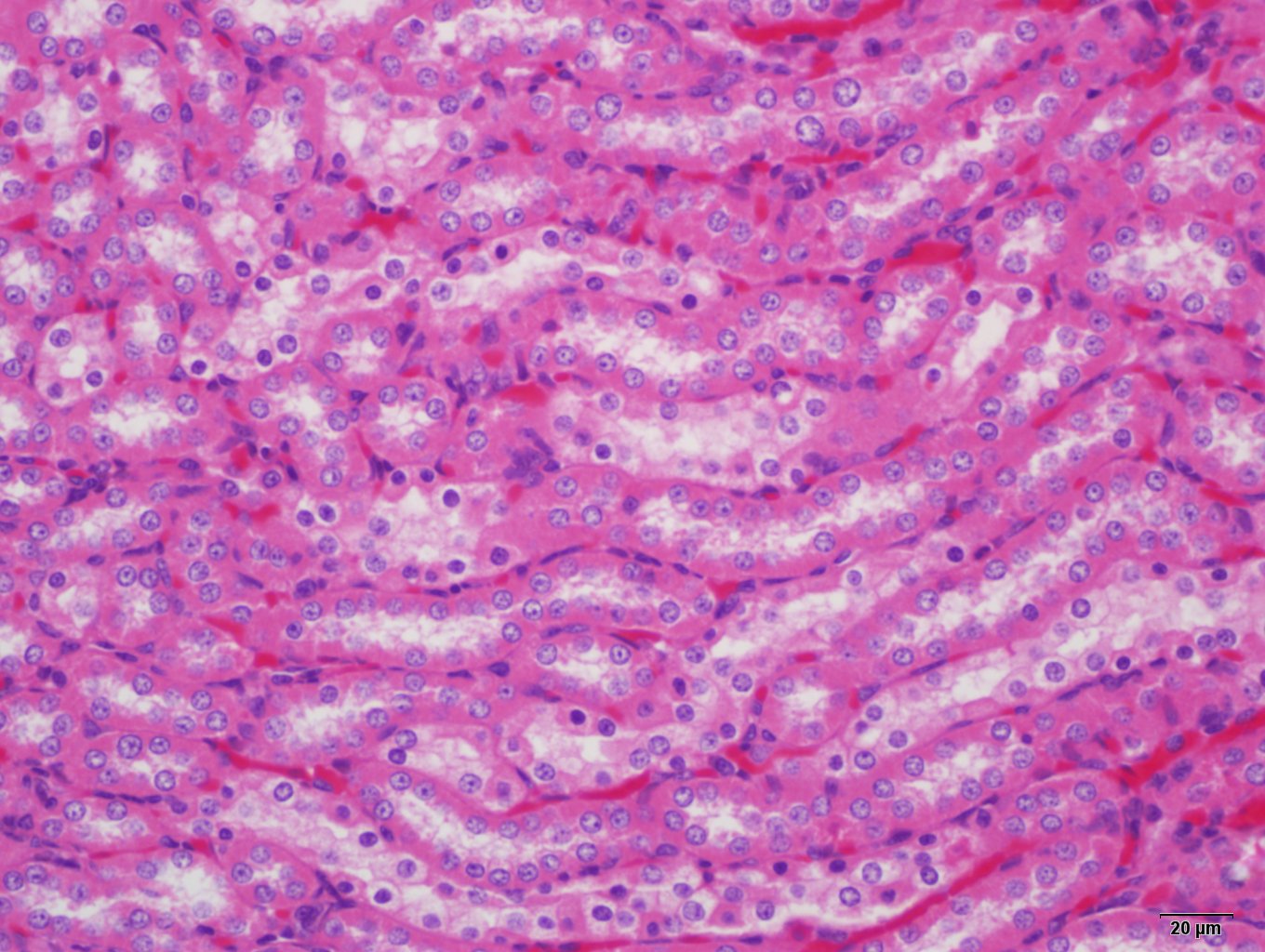

Supplement: Supplemental Information 20 [file peerj-08-8971-s020.jpg]

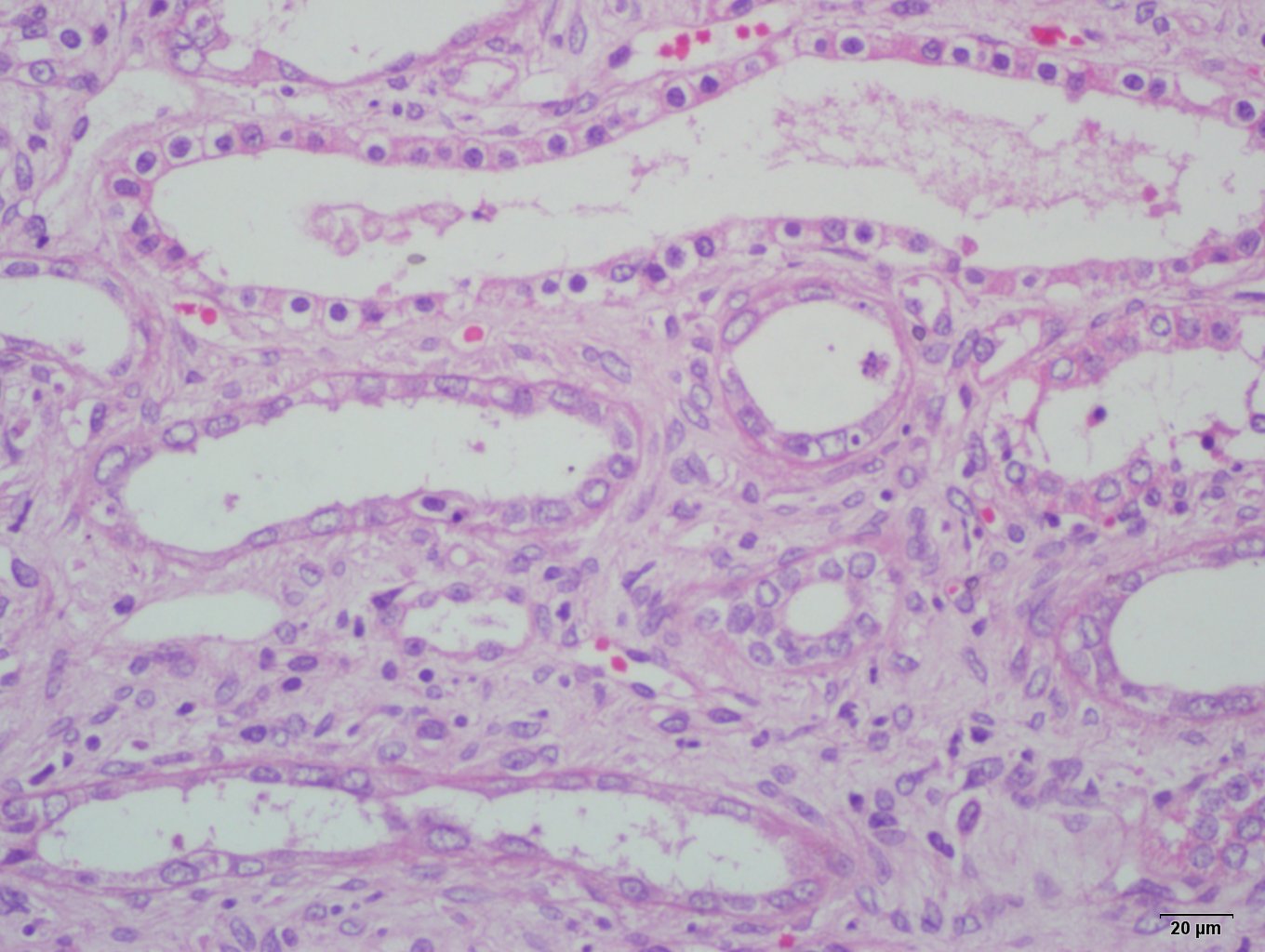

Supplement: Supplemental Information 21 [file peerj-08-8971-s021.jpg]

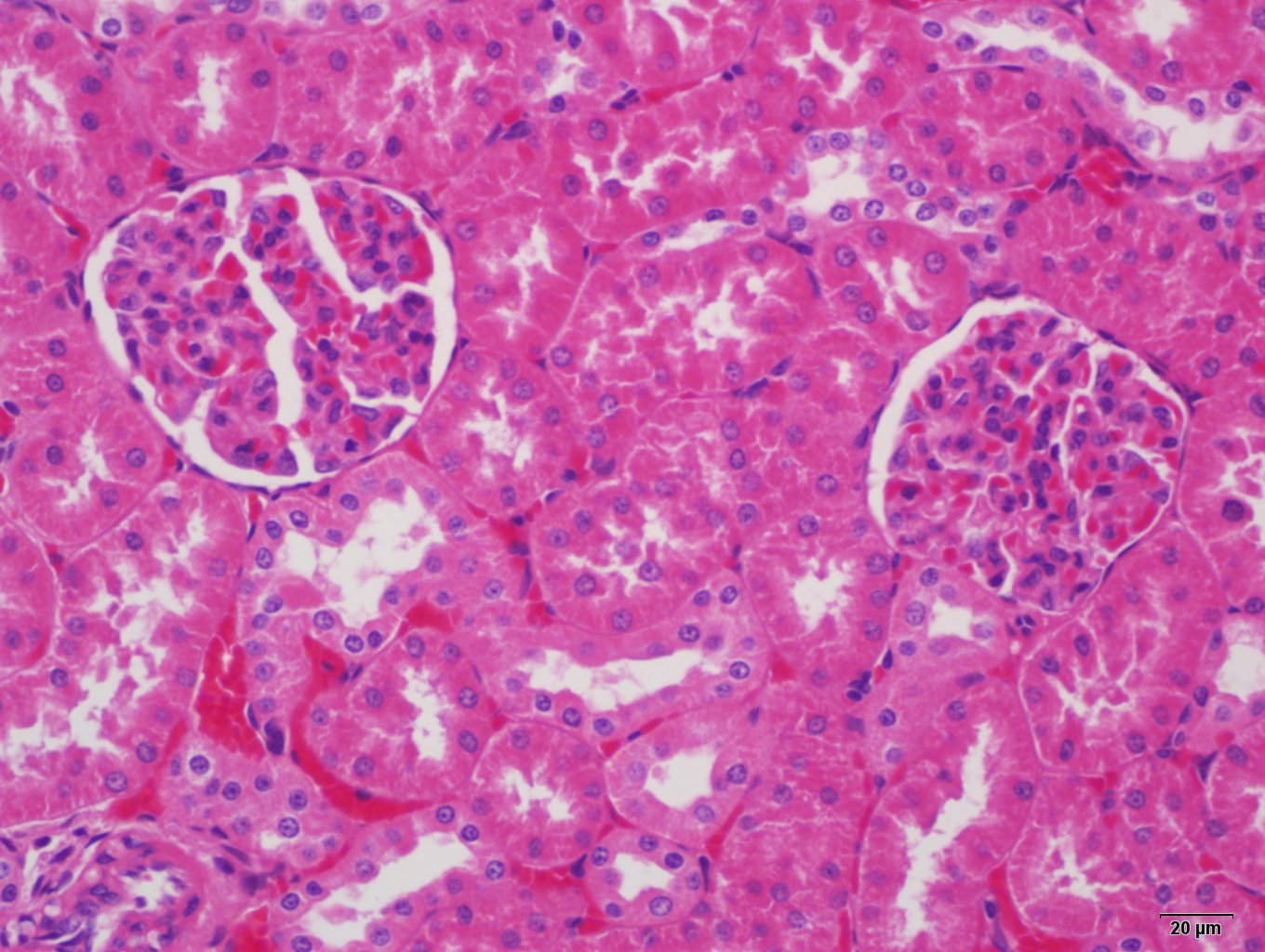

Supplement: Supplemental Information 22 [file peerj-08-8971-s022.jpg]
