# Supplementary material for: Uricase-deficient rat is generated with CRISPR/Cas9 technique
Source: PeerJ. 2020 Apr 27;8:e8971. doi: 10.7717/peerj.8971 (PMC7192158; doi:10.7717/peerj.8971)
Supplement: Supplemental Information 23 — Uox mRNA sequence spliced from Figs. 3A and 3B and their original raw data. [file peerj-08-8971-s023.doc]

Uricase mRNA of Uox^-/-^ rat (in red). The total sequence is uricase mRNA of wild type rat. Since exon 2, exon 3 and exon 4 (part) was deleted, the modified uricase mRNA is 944 bp rather than 1,359 bp.
